# Supplementary material for: Grapevine leaf MALDI-MS imaging reveals the localisation of a putatively identified sucrose metabolite associated to Plasmopara viticola development
Source: Front Plant Sci. 2022 Oct 10;13:1012636. doi: 10.3389/fpls.2022.1012636 (PMC9589281; doi:10.3389/fpls.2022.1012636)
Supplement: Supplementary file 1 [file DataSheet_1.pdf]

Supplementary material

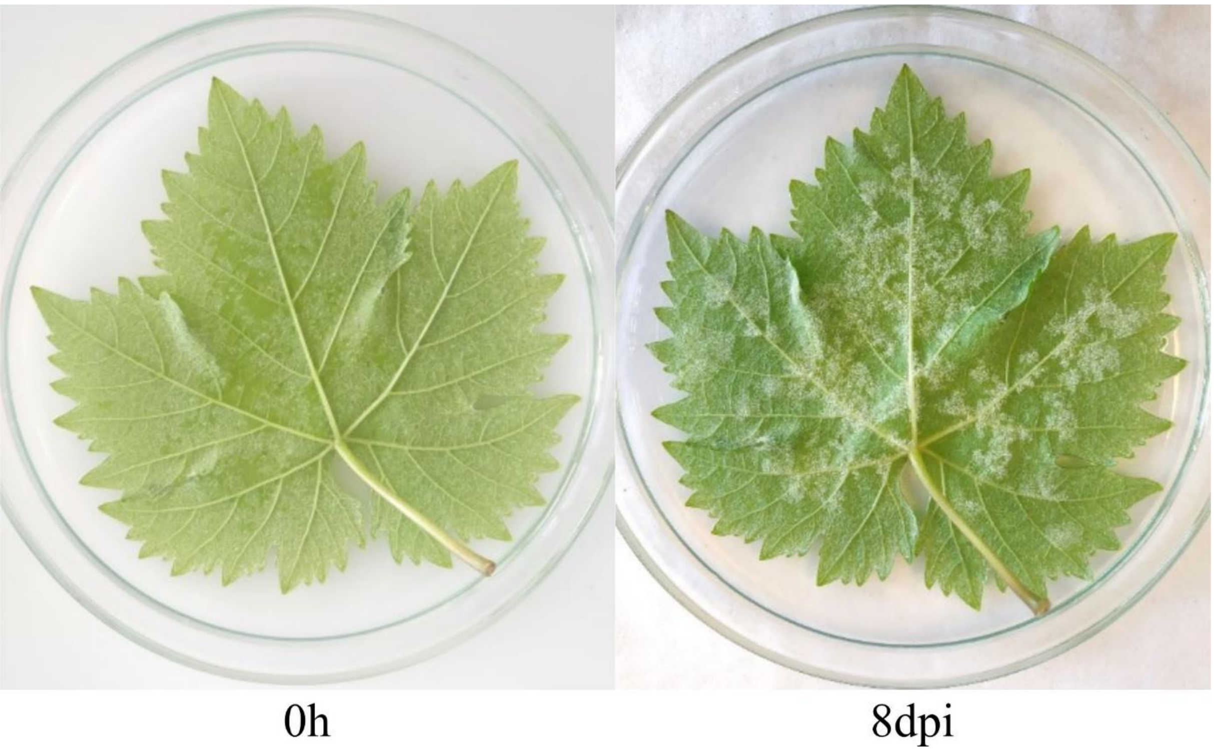

Supplementary Figure S1. *V. vinifera* cv Trincadeira phenotype leaves of non-infected (0 h) and 8 days after inoculation (8 dpi) with *P. viticola*.

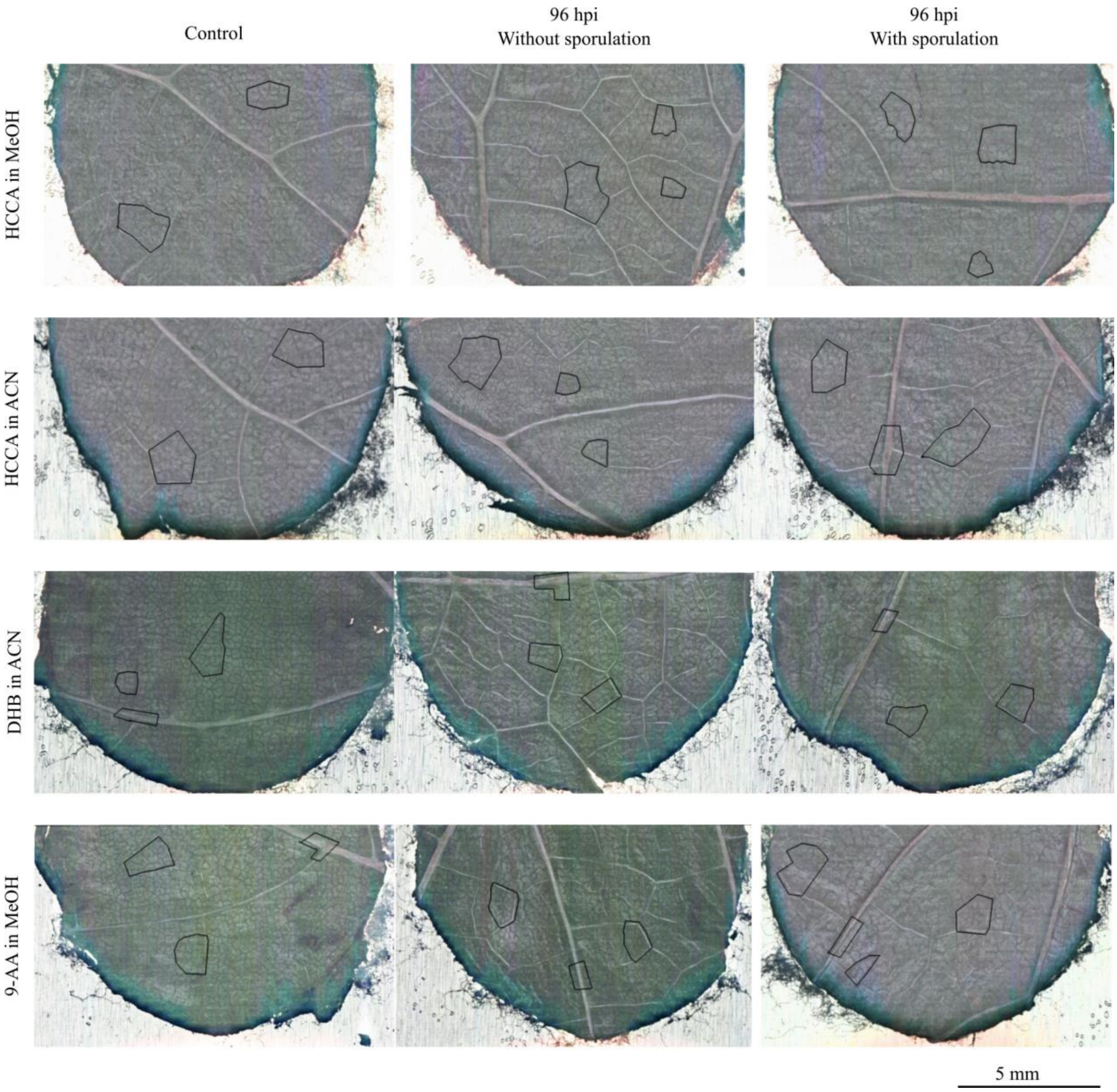

Supplementary Figure S2. Microscope images (10 x) of *Vitis vinifera* 'Trincadeira'. Areas marked with a black line were selected for MALDI-FT-ICR-MS analysis with different matrices and solvents.

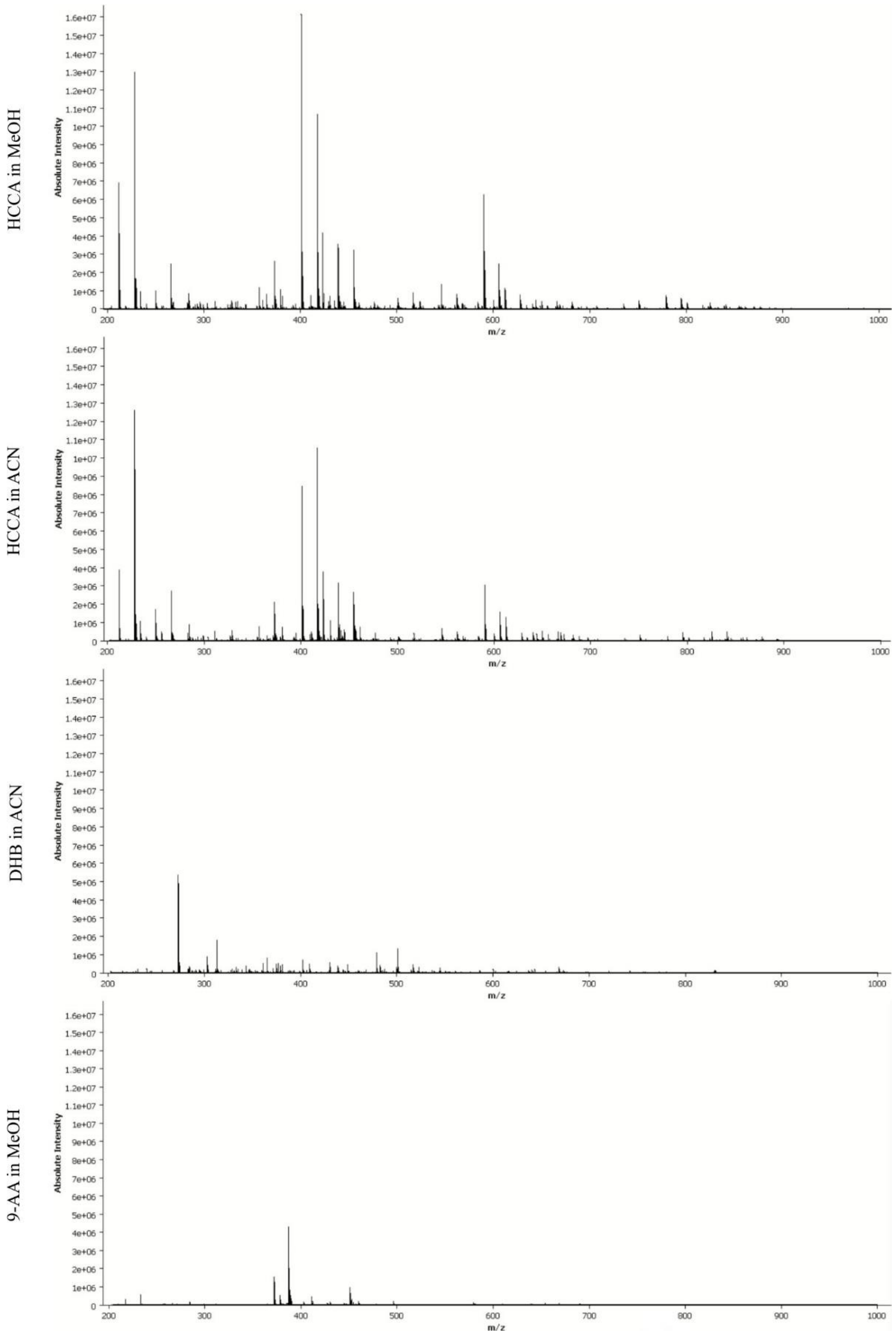

Supplementary Figure S3. MALDI-FT-ICR-MS mean mass spectra of *Vitis vinifera* 'Trincadeira' leaf discs (control, 96 hpi without visible *P. viticola* sporulation and 96 hpi with visible sporulation) in positive ionisation mode. Data was normalised by the total ion count.

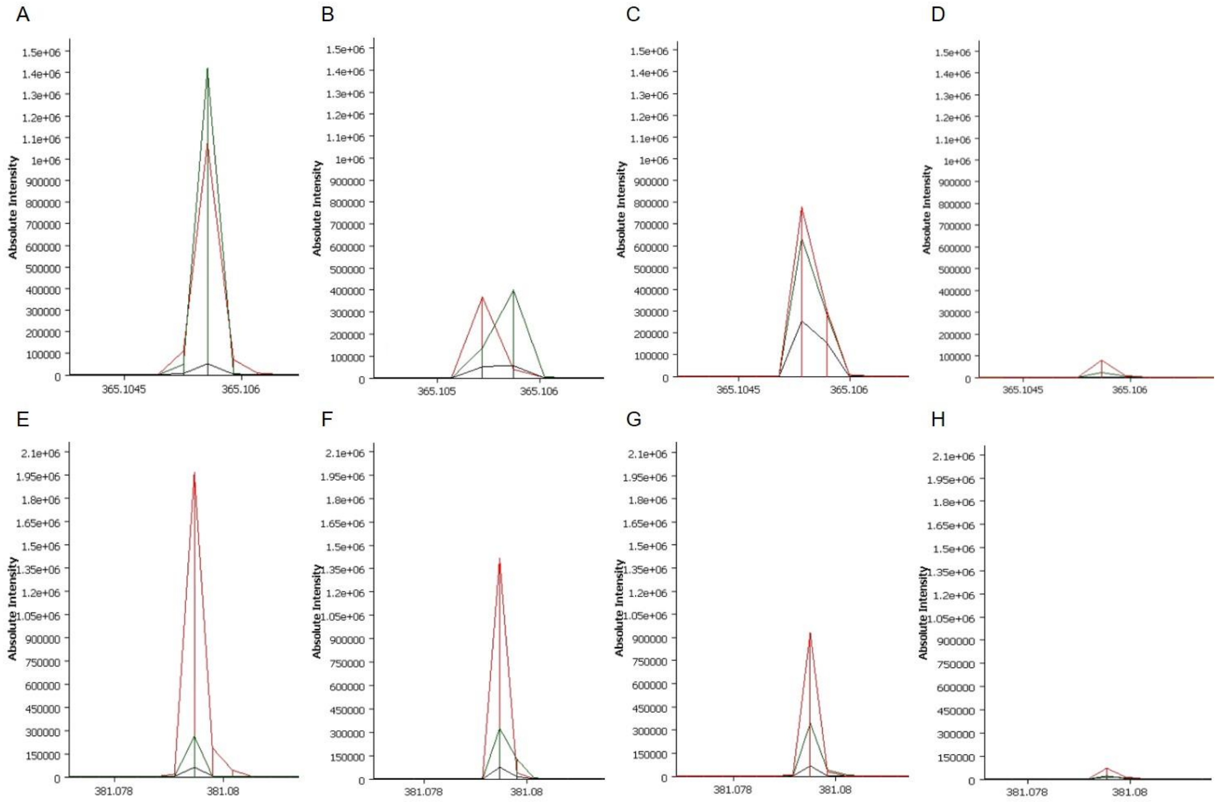

Supplementary Figure S4. MALDI-FT-ICR-MS average mass spectra obtained from *V. vinifera* ‘Trincadeira’ leaves for the putatively identified sucrose ions: *m/z* 365.105 $[M+Na]^+$ (**A**-**D**) and *m/z* 381.079 $[M+K]^+$ (**E**-**H**). **A** and **E**, HCCA matrix with MeOH; **B** and **F**, HCCA matrix with ACN; **C** and **G**, DHB matrix; and **D** and **H**, 9-AA matrix. Black, green and red spectra represent, respectively, controls, 96 hpi without visible *P. viticola* sporulation and 96 hpi with visible *P. viticola* sporulation. Data was normalised by the total ion count.

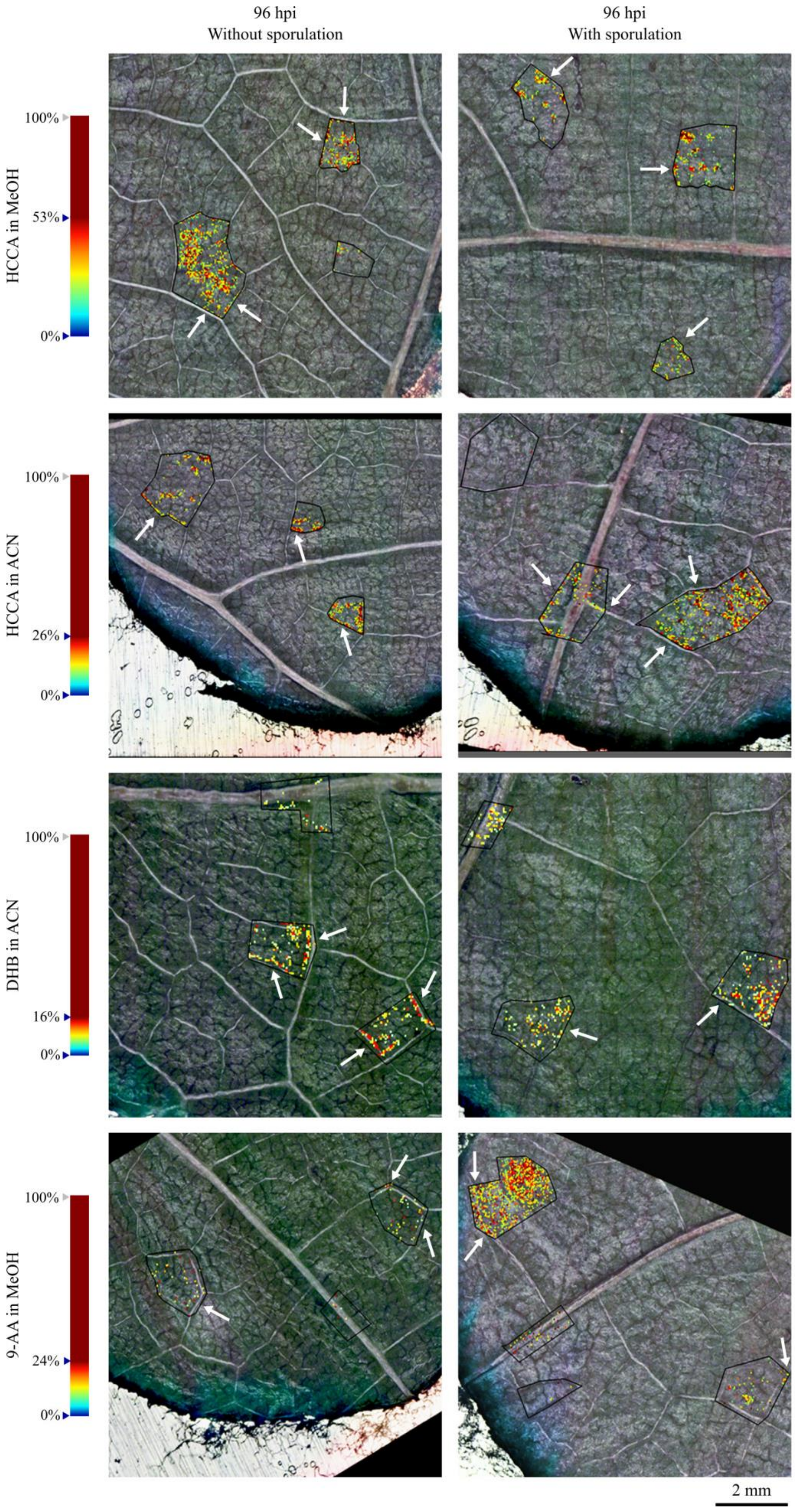
96 hpi
Without sporulation
96 hpi
With sporulation
HCCA in MeOH
100%
53%
0%
HCCA in ACN
100%
26%
0%
DHB in ACN
100%
16%
0%
9-AA in MeOH
100%
24%
0%
2 mm

Supplementary Figure S5. Reconstructed ion images of putatively identified sucrose (*m/z* 365.105, $[M+Na]^+$) in 96 hpi without visible *P. viticola* sporulation and 96 hpi with visible *P. viticola* sporulation. Leaf disc areas detected via MALDI–FT-ICR-MS imaging using HCCA matrix with MeOH and ACN, DHB matrix and 9-AA matrix. The colour scale indicates the absolute intensity of each pixel (arbitrary units). White arrows indicate the visible distribution of putatively identified sucrose along the leaf veins. Spatial resolution: 30µm.
